# Imparting Waterproofing Properties to Leather by Polymer Nanoemulsion Based on Long-Chain Alkyl Acrylate

**DOI:** 10.3390/ma16041464

**Published:** 2023-02-09

**Authors:** Liqiang Jin, Wenbin Xu, Hongmei Wen, Yulu Wang, Feifei Zhang

**Affiliations:** 1 Faculty of Light Industry, Qilu University of Technology (Shandong Academy of Sciences), Jinan 250353, China; 2 Key Laboratory for Green Technology of Leather Manufacture, China National Light Industry Council, Jinan 250353, China

**Keywords:** waterproof fatliquor, mini-emulsion polymerization, long-chain alkyl acrylate, reactive emulsifier

## Abstract

The demand for waterproof leather has been increasing, and environmentally friendly waterproof fatliquors have recently received increasing attention. In this work, two polymer nanoemulsions containing carboxyl groups were synthesized and used as waterproof fatliquors for chrome-tanned leather. First, a reactive emulsifier (C_12_-Na) was prepared using itaconic anhydride and lauryl alcohol. Subsequently, two polymer nanoemulsions were prepared through mini-emulsion polymerization with C_12_-Na as the emulsifier, 4,4′-azobis (4-cyanovaleric acid) as the initiator, and lauryl acrylate (LA)/stearyl acrylate (SA) as monomers; these were named PLA and PSA. PLA and PSA were characterized using FT-IR, a Zetasizer, and GPC. It was found that the critical micellar concentration (CMC) of C_12_-Na was 2.34 mmol/L, which could reduce the surface tension of water to 26.61 mN/m. The average particle sizes of PLA and PSA were 53.39 and 67.90 nm, respectively. The maeser flexes of leather treated with PLA and PSA were 13928 and 19492 at a 5% dosage, respectively, and the contact angles reached 148.4° and 150.3°, respectively; these values were both higher than for a conventional fatliquor. Compared with PLA, the leather treated with PSA exhibited better fullness, and tensile and tearing strength. The prepared nanoemulsions have prospective applications in leather manufacturing as waterproof fatliquors.

## 1. Introduction

Waterproofness is one of the most valuable performances of leather products. In recent years, the production and market share of waterproof leathers have continued to increase dramatically worldwide [1]. As a natural protein matrix, the leather material itself is hydrophilic. Leather waterproofness can be acquired by treating the leather with a waterproof fatliquor. In the leather-making process, waterproof fatliquors can impart leather with not only softness but also a certain degree of water resistance. Recently, more attention has been paid to the preparation and application of waterproof fatliquors [2,3,4].

Many chemicals have been developed as waterproof fatliquors, such as alkenyl succinic acid, chromium soaps, polysiloxanes, organic fluorine compounds, and amphiphilic polymeric fatliquors [5,6,7]. Among these chemicals, amphiphilic polymeric fatliquors have become a research hot spot due to their ability to integrate the retanning and fatliquoring process and shorten the processing time [8,9,10]. These polymers usually contain carboxyl groups and alkyl groups with long carbon chains. They demonstrate not only fatliquoring but also retanning abilities, and consequently render the leather with a high filling ability, softness, waterproofness, and low fogging. The polymeric fatliquors can be efficiently taken up by leather, demonstrating exhaustion rates of more than 90% [11]. After the fatliquoring process, the hydrophilic parts of the amphiphilic polymers can be capped by chromium, so as to maximize the water repellency of the leather.

Solution polymerization is a common method for preparing a waterproof polymer fatliquoring agent. The U.S. patent [12] reported the preparation of a polymeric water repellent fatliquor which was a copolymer of long-chain alkyl methacrylate and acrylic acid. This fatliquor could impart water repellency and dry-cleaning resistance to leather. Song et al. [13] developed a silicone-containing polymer fatliquor using octadecyl acrylate, methyl acrylate, and vinyltrimethoxysilane as reactive monomers and azo diisobutyronitrile as the initiator. The reaction was carried out in the toluene medium. Du et al. [14,15] used octadecyl acrylate and acrylic acid as reaction monomers, ethylene glycol monobutyl ether as the reaction medium, dodecyl mercaptan as the molecular mass modifier, and benzoyl peroxide as the initiator to prepare a series of water-repellent polymeric fatliquors and investigated the effect of polymer molecular mass on their application properties. In these research studies, organic solvents such as butanone, ethylene glycol monobutyl ether, and toluene were used as a polymerization reaction medium; they were harmful to the environment and to human health. The consumer pursuit of green and safety limited their practical application in leather processing.

Researchers attempted to synthesize a waterproof polymeric fatliquor in water. Zou et al. [16,17] reported the copolymerization of long-chain alcohol maleic monoester and acrylic acid in water to prepare multifunctional polymers with both retanning and waterproofing abilities. The crust leather showed a 2 h static water adsorption rate of 60% after it was fatliquored. In this reaction system, the copolymerization ability of the maleic monoester with other vinyl monomers was not strong enough to obtain amphiphilic polymers with a high molecular weight, which consequently resulted in a poor waterproof capacity. In addition, when ammonium persulfate was used as the initiator, sulfate groups were introduced at the end of the polymer chains due to the decomposition of the initiator, which further decreased the waterproof ability of the obtained product because the sulfate group was hydrophilic and not easily capped by chromium salt. Yu et al. [11] prepared an amphiphilic polymer fatliquor via the ternary emulsion copolymerization of two hydrophobic monomers, octadecyl acrylate and dodecyl, and one hydrophilic monomer, acrylic acid, in water. Lauryl sodium sulfate (SDS) was used as the emulsifying agent, cyclodextrin was used as the solubilizing carrier of long alkyl chain acrylates, and ammonium persulfate was used as the initiator. The performance of the obtained product was evaluated. The use of SDS and ammonium persulfate as emulsifier and initiator, respectively, led to a decrease in waterproofing due to their hydrophilicity, so it is necessary to identify a new initiation and emulsification system.

In order to overcome the current problems with the preparation of waterproof fatliquors, we developed a new strategy for preparing a waterproof polymeric fatliquor via mini-emulsion polymerization. Firstly, a reactive surfactant based on itaconic acid, C_12_-Na, was synthesized and used as the emulsifier. Next, two polymer nanoemulsions (PLA/PSA) were prepared through mini-emulsion polymerization with C_12_-Na as the emulsifier, 4,4′-azobis (4-cyanovaleric acid) as the initiator, and lauryl acrylate/stearyl acrylate as the monomer. Lauryl acrylate and stearyl acrylate are widely used to prepare waterproof fatliquors because their long alkyl chains may lubricate collagen fibers and provide the leather with hydrophobic properties. The reasons for designing this synthesis approach are based on the following points: 1) mini-emulsion polymerization is a robust and environmentally friendly method that can prepare a high-molecular-weight polymer by using water as the dispersed phase. The particle size of the emulsion can be preset by adjusting the amount of emulsifier [18,19]; 2) for a traditional emulsion, the emulsifier could fall off the surface of the emulsion particles while in use. It could preferentially diffuse to the surface of the collagen fibers and occupy the reaction sites due to its smaller size, thus affecting the binding of the emulsion particles with the fibers. We chose C_12_-Na as the reactive emulsifier because it can be anchored to the particle surface by the polymerization reaction and would not affect the binding between the polymer and collagen. In addition, this reactive emulsifier, which contains carboxyl groups, could be largely responsible for the high affinity of the polymers for chromed collagen as well as their solubility in water; and 3) in the polymerization process, with the decomposition reaction of the initiator of 4,4′-azobis (4-cyanovaleric acid), the carboxyl group was introduced into the polymer chains, which are easily capped by chrome salt without affecting the water resistance of the product. In this work, the preparation process of the reactive surfactant and polymer nanoemulsions was described in detail, the obtained reactive surfactant and polymer nanoemulsions were characterized, and their performance as waterproof fatliquors were also investigated.

## 2. Materials and Methods

### 2.1. Materials

Technical-grade lauryl acrylate (LA) and stearyl acrylate (SA) were provided by Shandong Leaning Chemicals. Co. Ltd., Yishui, China. Analytical-grade lauryl alcohol and sodium hydroxide (NaOH) were purchased from Sinopharm Chemical Reagent Co., Ltd., Shanghai, China. Itaconic anhydride and 4,4′-Azobis (4-cyanovalic acid) (ACPA, initiator) were purchased from Shanghai Macklin Biochemical Co., Ltd., Shanghai, China. Fatliquor SS (a sulfonated, rapeseed oil fatliquor), shaved, wet-blue cattle leather, and other chemicals were kindly provided by Luri Junda Leather Co. Ltd., Jinan, China.

### 2.2. Synthesis of Reactive Emulsifier

**Figure 1:**
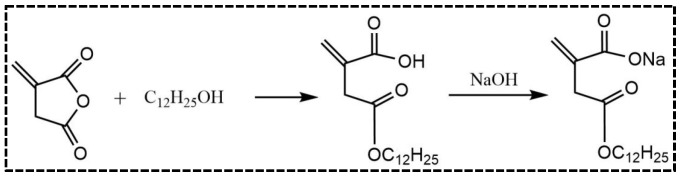
Synthesis of reactive emulsifier C_12_-Na.

The preparation procedure for the reactive emulsifier is shown in Figure 1. Firstly, a mixture of lauryl alcohol (1 mol) and itaconic anhydride (1 mol) was stirred at 80 °C for 4 h in a 250 mL, four-neck, round-bottom flask with a reflux condenser. It was then cooled to room temperature. The product was recrystallized with absolute ethanol to obtain lauryl itaconic acid monoester (LIA) with a yield of over 90%. Finally, LIA was dissolved in water and neutralized with a sodium hydroxide solution. After freeze-drying, the reactive emulsifier (C_12_-Na) was obtained.

### 2.3. Synthesis of Polymer Nanoemulsions

**Figure 2:**
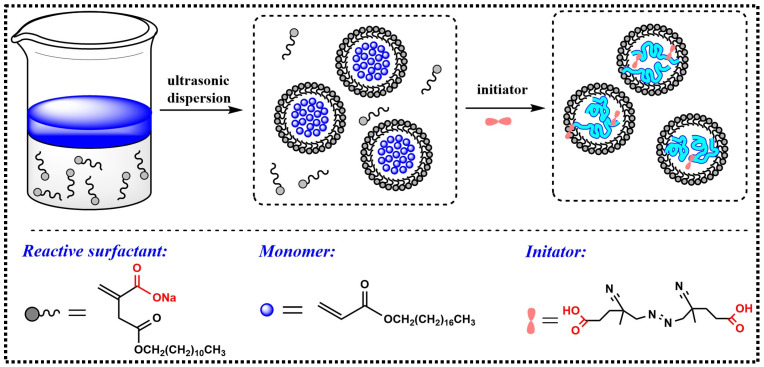
The synthetic route of polymer emulsion.

The nanoemulsions were prepared via mini-emulsion polymerization with a solid content of approximately 20%. The preparation route is shown in Figure 2. The monomer LA or SA was firstly added to the aqueous C_12_-Na solution and agitated at 30 °C for approximately 30 min. The resultant emulsion was sonicated using a BILON-500 Ultrasonic signal generator (Bilon Instrument Co. Ltd. Shanghai, China) at a 90 W intensity for 30 min to obtain the mini pre-emulsion.

The polymerization was carried out in a stirred, 500 mL flask fitted with an overhead condenser and two feed funnels. The temperature of the reactor was maintained at 70 °C by partially immersing the reactor in a thermostated water bath. A certain amount of water was first charged into the reactor and heated under stirring. The obtained mini pre-emulsion and the 4′-Azobis (4-cyanovalic acid) solution were then added dropwise into the reactor within 150 min. The temperature was kept constant for 120 min after the feeding was finished. The two polymer nanoemulsions, which were named PLA and PSA respectively, were obtained according to the polymerization recipe provided in Table 1.

### 2.4. Application of Polymer Nanoemulsions in Fatliquoring

The fatliquoring was carried out in a GSD400-4 rotary drum (Xinda Light Industry Machinery Co., Ltd. Wuxi, China). After been rewetted, retanned, and neutralized as usual, wet-blue leather samples from cattle were divided into two parts along the backbone. The left half was fatliquored with sulfonated oil, while the right half was treated with PLA and PSA. The untreated leather was used as the blank. The detailed processing conditions are listed in Table 2.

### 2.5. Characterization

#### 2.5.1. FT-IR

The FT-IR spectrum was determined using a Nicolet 10 infrared spectrometer (Shimadzu, Kyoto, Japan). The scanning frequency range was 4000–400 cm^−1^.

#### 2.5.2. H NMR

The ^1^H NMR spectra of the synthesized C_12_-Na were recorded using an Avance II-400 spectroscopy (Bruker Co., Ltd., Bremen, Germany), using deuterated chloroform as the solvent and tetramethylsilane (TMS) as the internal standard.

#### 2.5.3. Surface Properties of C_12_-Na 

The surface tension (γ) of the freshly prepared C_12_-Na solutions was measured at 25 ± 0.5 °C by a BZY-4B automatic surface tension tester (Hengping Instrument and meter factory, Shanghai, China).

#### 2.5.4. Emulsifying Capacity of C_12_-Na

The emulsification capacity of the C_12_-Na solutions (0.1wt%) was determined by a water segregating method, according to the literature [20].

#### 2.5.5. Particle Size and Zeta Potential Measurement

The particle sizes and zeta potential of PLA and PSA were measured using a zeta potential and nanoparticle size analyzer (Nano-ZS90, Malvern, UK).

#### 2.5.6. Stability

The centrifugal stability of PLA and PSA were determined by centrifugation at 4000 ppm for 30 min using a TGL-16 tabletop centrifuge (Anting Scientific Instrument Factory, Shanghai, China). The dilution stability and the stability to acid, alkali, chromium salt, and vegetable tannin were conducted according to the QB/T 2158-1995 standard.

#### 2.5.7. Absorption of Polymeric Emulsion into the Chromed Leather

The absorption rate of the polymeric emulsion into the leather can be calculated by measuring the content of the total organic carbon (TOC) in float before and after fatliquoring at a pH of 4.0–4.5 using a Multi N/C 2100S total organic carbon/total nitrogen analyzer (Analytik, Jena, German). Three leather samples were tested after being processed with PLA and PSA, respectively, and the average values were reported.

#### 2.5.8. Physical and Mechanical Properties

The thickness increment ratio of the fatliquored leather was measured using a GT-313-A thickness gauge (Gotech, Dongguan, China). The softness of the leather was measured using a GT-303 softness tester (Gotech, Dongguan, China). The tensile and tearing strength of the leather before and after fatliquoring were tested using a XWN-20 microcomputer control tensile tester (Kexin test Instrument Co., Ltd., Changchun, China). The resistance to yellowing of the resultant leather was assessed according to the QB/T 4672-2014. Three leather samples were tested to obtain the mean values of the physical properties.

#### 2.5.9. Contact Angle Measurements

The contact angle (CA) was measured using an optical contact angle meter system (DSA25, KRUSS, Hamburg, Germany) by dropping 20 µL of deionized water onto the leather surface at room temperature. The final values was the average, determined over five different locations for each sample.

#### 2.5.10. Water Penetration Test

The maeser flex of the leather was detected using a GT-7071-DW Maeser Tester (Gotech, Dongguan, China). The experiments were conducted in triplicate, and the average value and standard deviation were calculated.

#### 2.5.11. Scanning Electron Microscope (SEM)

The microstructures of the treated leather fibers were observed with a scanning electron microscope (G6, Phenom Scientific, Eindhoven, The Netherland). The micrographs of the cross-sections of the leather samples were obtained at a magnification of 250×.

## 3. Results and Discussion

### 3.1. The Preparation of C_12_-Na

**Figure 3:**
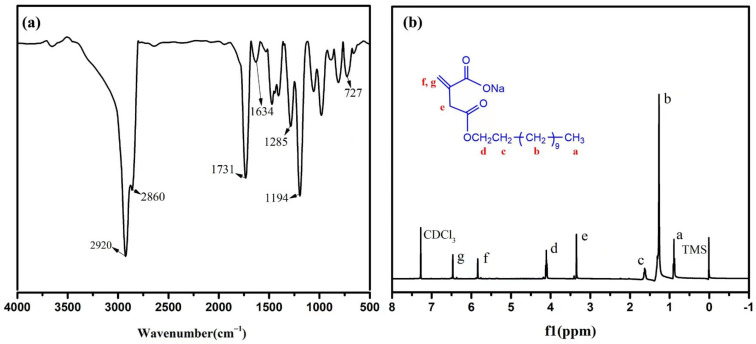
(**a**) FT-IR and (**b**) ^1^H NMR of C_12_-Na.

FT-IR and ^1^H NMR were used to characterize the structure of the reactive emulsifier, C_12_-Na. The FT-IR spectra of C_12_-Na are represented in Figure 3a. The bands at 2916 cm^−1^ and 2851 cm^−1^ were due to the presence of -CH_3_ and -CH_2_ in the C_12_-Na. The band at 1726 cm^−1^ was assigned to the stretching vibrations of the -C=O. The band at 1634 cm^−1^ was due to the -C=C- stretching vibrations, the band at 1166 cm^−1^ was assigned to the -C-O-C- stretching vibrations, and the band at 720 cm^−1^ was attributed to the bending vibration band of -(CH_2_) n. The ^1^H NMR of the C_12_-Na is shown in Figure 3b: σ 0.87~0.89 (3H, a-H); σ 1.25~1.30 (18H, b-H); σ 1.58~1.64 (2H, c-H); σ 3.34 (2H, e-H); σ 4.08~4.11 (2H, d-H); σ 5.83 (1H, f-H); and σ 6.46 (1H, g-H). The results confirmed that the structure of the prepared products were the target compounds [21].

**Figure 4:**
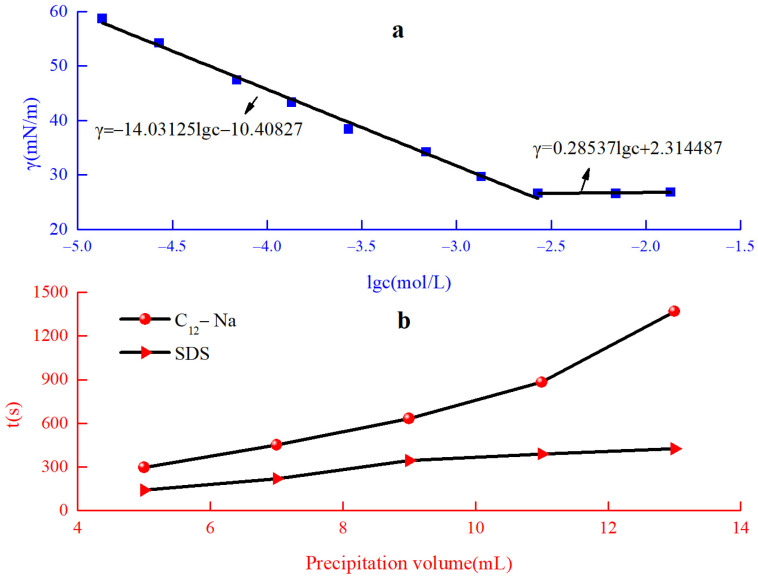
(**a**) The surface tension and (**b**) the emulsification ability of C_12_-Na.

The surface tension of the C_12_-Na was measured to evaluate its surface activity. The surface tension of the C_12_-Na solution as a function of the logarithm of surfactant concentration is shown in Figure 4a. The critical micellar concentration (CMC) of the C_12_ -Na could be obtained from the intersection of two lines after linear fitting. The prepared reactive emulsifier, C_12_-Na, demonstrated high surface activity with a CMC of 2.34 mmol/L, and the surface tension at CMC was 26.61 mN/m. The emulsifying capacity can be evaluated by the time it takes to release the same volume of liquid using a water-segregating method [22]. The longer the time needed to precipitate the same volume, the better the emulsification performance of the surfactant is. The emulsification power of C_12_-Na and SDS are shown in Figure 4b. When the same volume of water was precipitated, the time required for C_12_-Na was greater than that for SDS. This indicates that the emulsification power of C_12_-Na was superior to the emulsification power of SDS.

### 3.2. Characterization of PLA and PSA 

**Figure 5:**
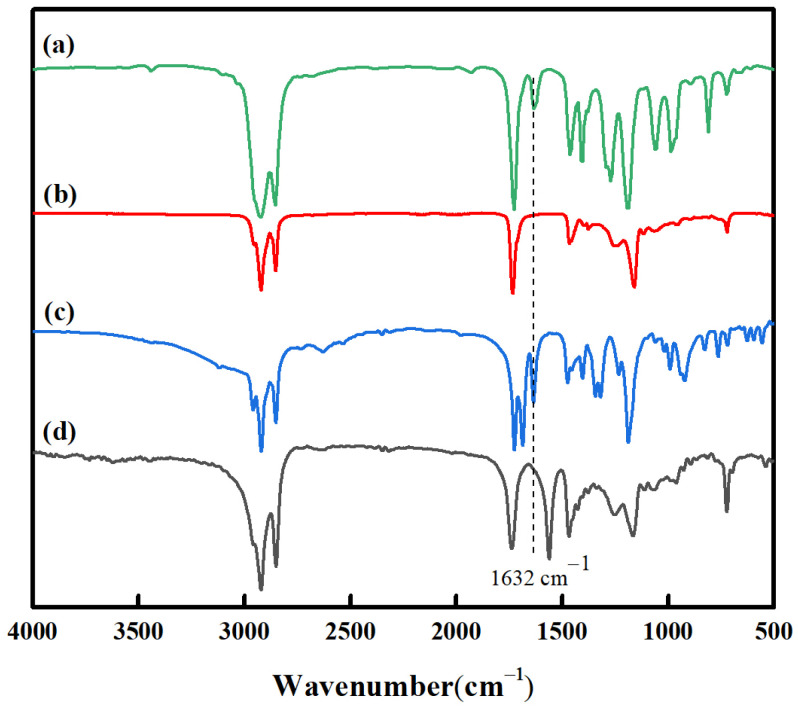
FTIR spectra of LA (**a**), PLA (**b**), SA (**c**), and PSA (**d**).

The FT-IR spectra of LA, PLA, SA, and PSA are shown in Figure 5. The bands at 2926 and 2824 cm^−1^ were from the stretching vibrations of -CH_3_ and -CH_2_. The bands at 1732 cm^−1^ were attributed to -C=O stretching vibrations. The bands at 1401 cm^−1^ and 1454 cm^−1^ were due to -CH_3_ bending vibrations, while the band at 1161 cm^−1^ was attributed to the -C-O-C stretching vibration. In the spectra of LA (Figure 5a) and SA (Figure 5c), the bands at 1634 cm^−1^ were assigned to the characteristic absorption of -C=C. This peak was absent in the spectra of PLA (Figure 5b) and PSA (Figure 5d), which suggests that the acrylic monomers reacted completely and the polymers were formed through the addition across the vinyl bond.

**Figure 6:**
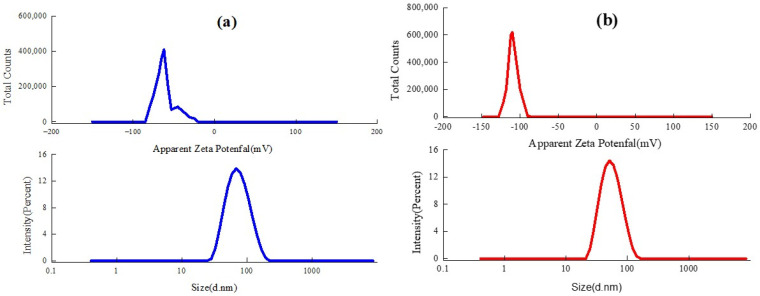
Zeta potential and particle size of PLA (**a**) and PSA (**b**).

The zeta potential, particle size, and distribution of the PLA and PSA are demonstrated in Figure 6. The zeta potential is an indicator of the charges carried by particles suspended in water, which measures the difference in electrical charge between the dense layer of ions surrounding the particle and the bulk of the suspended fluid [23]. The zeta potentials of the PLA and PLA were −110 mV and −62 mV, respectively, suggesting that both nanoemulsions were quite stable in the water due to the electric repulsion. The particle size of an emulsion directly determines its ability to penetrate the leather fibers. The smaller particle size allows the fatliquor to permeate and disperse into the dense collagen fibers more easily. The average particle sizes of the PLA and PSA were found to be 53.39 nm and 67.90 nm, respectively. The distribution of the polymeric particles was very homogenous, with polymer dispersity indexes (PDIs) of 0.124 and 0.143, respectively. According to the theoretical model reported by Reich, the pore sizes between collagen fibrils are about 100 nm. Both sizes were smaller than the pore sizes [24], which could facilitate their infiltration into the inner part of the collagen fibers, consequently improving their application performance.

### 3.3. The Stability of PLA and PSA

**Figure 7:**
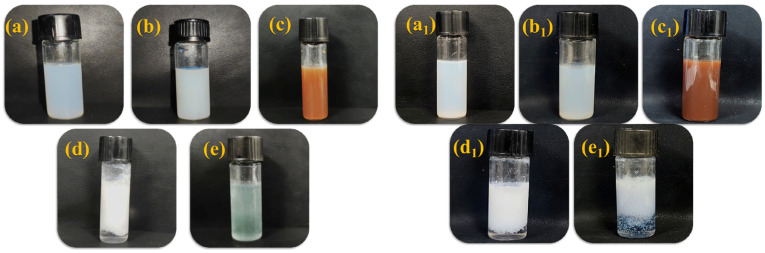
Stability of PLA (**left**) and PSA (**right**): (**a**,**a1**) 1:9 dilution, (**b**,**b1**) centrifugal, (**c**,**c1**) tanning extract, (**d**,**d1**) acids, and (**e**,**e1**) chromium salt.

The results of emulsion stability test of PLA and PSA are shown in Figure 7. As is shown in Figure 7, The PLA and PSA both exhibited a high dilution stability (Figure 7a,a1) and centrifugation stability (Figure 7b,b1). The high stability may result from their high zeta potential values and nanosized particle diameters. While PLA and PSA were stable in vegetable tannin (Figure 7c,c1), they coagulated in acid (Figure 7d,d1) and chrome salt (Figure 7e,e1). In the acid, the deionization of carboxyl groups was inhibited, resulting in a low hydrophilicity of the polymer emulsions. The carboxyl groups in the polymer emulsions could coordinate with the chromium salt, and this consequently led to coagulation. Therefore, the PLA and PSA could be fixed by acids and chromium salt at the end of the fatliquoring process to maximize their waterproofing abilities.

### 3.4. The Absorption of PLA and PSA into the Chromed Leather

**Figure 8:**
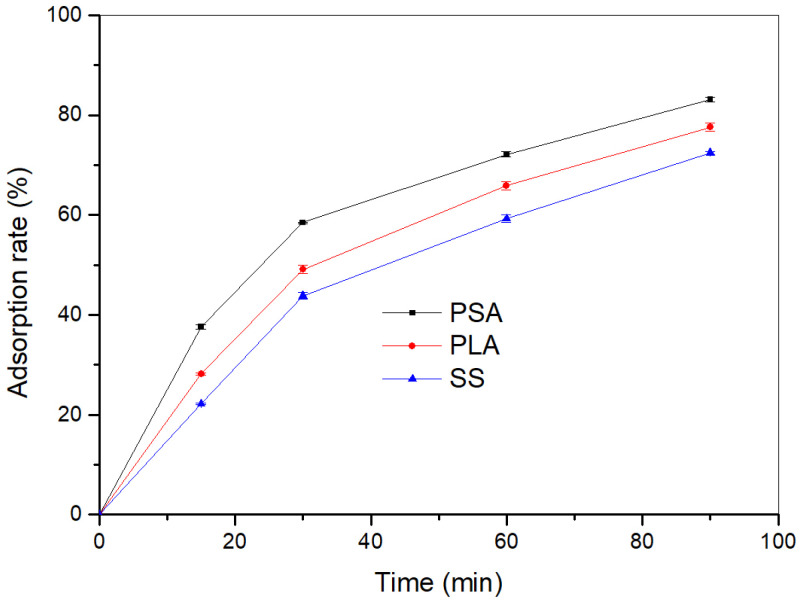
Absorption rate of fatliquors with running time.

The absorption rate of PLA and PSA was evaluated by measuring the TOC content of float before and after fatliquoring at a pH of 4~4.5. A plot of the absorption rate of the fatliquoring agents versus running time is shown in the Figure 8. As is shown in Figure 8, the absorption ratios of the PLA, PSA, and sulfonated oil (SS) all increased with the running time. After 90 min, the absorption rates reached 92.62%, 98.13%, and 87.47%, respectively. The absorption ratios of PLA and PSA were higher than that of the conventional fatliquor, SS. This is because the carboxyl groups in the PLA and PSA could coordinate with chromium, which imparted them with a better affinity for the chrome-tanned leather; therefore, they were absorbed more into the leather.

### 3.5. Physical Properties and Morphology of the Fatliquored Leather

Table 3 shows the physical and mechanical properties of the leathers treated with different fatliquors, including the thickness increment ratio, softness, yellowing resistance, tensile strength, and tearing strength. The thickness increment ratio is usually used to evaluate the filling ability of the chemicals. SS gave a thickness increment ratio of −1.71%, lower than the thickness before the fatliquoring, indicating its poor filling ability. The thickening rates of the leathers treated with PLA and PSA were much higher than that of leather treated with the SS. This result showed that the two prepared nanoemulsions could impart leather with an excellent fullness. Compared with PLA, PSA demonstrated a better filling ability due to its longer alkyl chain. The tensile and tearing strength of the leather treated with PLA and PSA were better than those of SS, which should be attributed to the improvement in the cross-link density of the fatliquored leather. PLA and PSA contain a large number of carboxyl groups, which easily coordinate with the chromium in collagen fibers, thus forming new cross-linking networks and enhancing the tensile and tearing strength.

**Figure 9:**
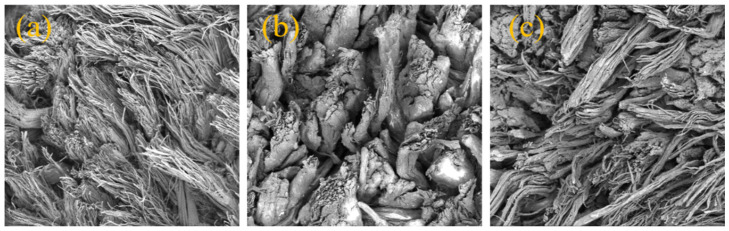
SEM images of the leather fibers: SS (**a**), PLA (**b**), and PSA (**c**).

Softness is also an important index, showing the softening ability of a fatliquor [25]. The softness of the untreated leather (blank) was 0.86 mm, while the softness of leather treated by PLA, PSA, and SS was 4.84, 5.94, and 9.34, respectively. The softness of the leather was improved after treated with PLA and PSA due to the lubricating action of the hydrocarbon chain in the polymer molecular structure. However, the softness of leather treated with PLA and PSA was lower than that of the SS-treated leather. This may be because SS has a smaller molecular mass and could therefore penetrate not only the collagen fibrils but also the collagen microfibrils [24]. SEM could be used to look deeply into the hierarchy structure of the leather. Figure 9 shows the SEM images of the cross-sections of leathers treated with SS, PLA, and PSA. The collagen fibers treated with SS were effectively separated, while those fatliquored with PLA and PSA were less dispersed. The PLA and PSA mainly filled in between the collagen fiber bundles and acted as a filler, thus obtaining a lower softness than the conventional fatliquor, SS.

The yellowing resistance value indicates the color stability of the leather under light. The higher the yellowing resistance value, the more stable of the leather color is [26]. As is shown in Table 3, the grade of yellowing resistance of the leather fatliquored by SS was 1.5, while the grades of yellowing resistance were 4.5 and 4 for the leather samples treated with PLA and PSA, respectively. Since the maximum yellowing resistance grade was 5, the PLA- and PSA-treated leather exhibited a very high color stability. The polymeric nanoemulsions could render the leather with a high light resistance, which would benefit their practical application.

### 3.6. Waterproofness of the Fatliquored Leather

**Figure 10:**
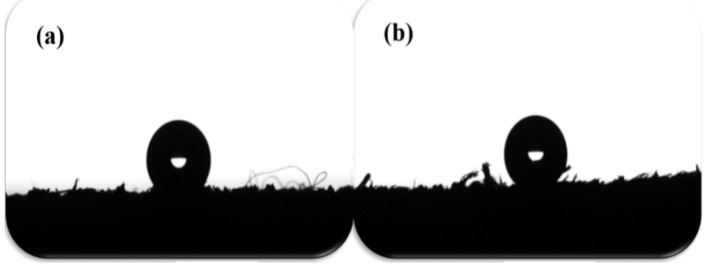
The water contact angle of the leather treated with PLA (**a**) and PSA (**b**).

The hydrophobic performance of solid surfaces can be characterized by the contact angle of water on its surface. Figure 10 shows the contact angle of the leather fatliquored with PLA and PSA. The higher the contact angle, the better the hydrophobicity [27]. As can be seen from Figure 10, the contact angles of water on the leather surfaces treated with PLA and PSA were 148.4° and 150.3°, respectively, indicating that super-hydrophobic surfaces were obtained after the treatments with PLA and PSA.

The maeser flex is an important index for evaluating the waterproofness of leather products. It is well-known that leather with a maeser flex higher than 15,000 can meet the market requirements for waterproof leather. The maeser flex of the leather treated with different fatliquors is shown in Table 4. As can be seen from Table 4, the maeser flex of the leather fatliquored by SS was 205, while the maeser flexes of the leather fatliquored by PLA and PSA were 13,928 and 19,492, respectively: much higher than that of SS. It was reported that the maeser flex of a leather sample fatliquored with a polymeric fatliquor prepared using maleic diester was 642 [17], much lower than those of the PLA and PSA. The maeser flexes of the leather sample fatliquored by the commercial waterproof fatliquor, WP, was 17,442 at a dosage of 5% [28]; this was prepared using ethylene glycol monobutyl ether as the solvent. It could be concluded that the prepared nanoemulsions, especially PSA, exhibited a highly waterproof performance and could be used in the production of water-resistant leather.

## 4. Conclusions

The present study developed a new method of preparing waterproof fatliquors. Two polymer nanoemulsions containing carboxyl groups were prepared through mini-emulsion polymerization with dodecyl itconate monoester as the emulsifier, 4,4′-azobis (4-cyanovaleric acid) as the initiator, and long chain acrylate as the monomer in the water medium. It was found that the polymer emulsions were nanoparticles with a uniform size that were sensitive to acid and chrome salt. The synthesized polymer nanoemulsions were used as waterproof fatliquors for chrome-tanned leather. The application results show that the obtained polymer emulsions demonstrated a high affinity for chromed leather. After being fatliquored with PLA and PSA, leather surfaces with higher hydrophobicities could be obtained. The maeser flex of leather treated with PSA could meet the requirements for commercial waterproof leather. Compared to PLA, PSA exhibited better physical properties and waterproofness due to its longer hydrophobic chain. Being solvent-free and environmentally friendly, the prepared polymer nanoemulsions are suitable for the production of ecological, water-resistant leather.

## Figures and Tables

**Table 1:** Recipe of PLA and PSA nanoemulsions.

Ingredients (g)	PLA	PSA
Deionized water	100	100
C_12_-Na	1	1
Lauryl acrylate (LA)	18	0
Stearyl acrylate (SA)	0	18
Azobis (4-cyanovalic acid)	1	1
Sodium bicarbonate	0.25	0.25

**Table 2:** The application formulation of polymer nanoemulsion.

	Product	Quantity (%) ^a^	Temperature (°C)	Time (min)	pH
Neutralisation	Water	200	30	40	
	Sodium formate	2			
	Sodium bicarbonate	1.2		90	6~6.5
Washing					drain
Fatliquoring	Water	150	50		
	sulfonated oil or nanoemulsion	5 ^b^		90	
	Formic acid	2		30	3.8~4.0 drain
Fixation	Water	200	30		
	Chromium salt	2		30	drain
Washing				10	

^a^: based on wet-blue weight. ^b^: based on the solid content.

**Table 3:** Physical and mechanical properties of the fatliquored leather.

Scheme	Thickness Increment Rate (%)	Tensile Strength (MPa)	Tearing Strength (N/mm)	Softness (mm) ^a^	Yellowing Resistance
PLA	14.90 ± 0.92	9.30 ± 0.12	80.42 ± 4.9	4.84 ± 0.013	4.5
PSA	24.41 ± 2.1	11.23 ± 1.2	83.58 ± 5.1	5.94 ± 0.013	4
SS	−1.71 ± 0.22	8.68 ± 0.57	34.76 ± 4.2	9.34 ± 0.066	1.5

^a^, the softness of the untreated leather blank was 0.86 mm.

**Table 4:** The water penetration test of leather treated with different fatliquors.

Sample	Maeser Flexes
PLA	13928 ± 578
PSA	19492 ± 784
Sulfonated oil SS	205 ± 32

## Data Availability

Not applicable.
